# Endobronchial Ultrasound Diagnosis of a Malignant Superior Vena Cava Tumor Thrombus Extending Into the Right Atrium: An Unusual Cause of Recurrent Syncope

**DOI:** 10.7759/cureus.31704

**Published:** 2022-11-20

**Authors:** Larissa Check, Ariba Naz, Caleb Scott, Richard Duff

**Affiliations:** 1 Internal Medicine, Grand Strand Medical Center, Myrtle Beach, USA; 2 Pathology, Grand Strand Medical Center, Myrtle Beach, USA; 3 Pulmonary and Critical Care Medicine, Grand Strand Medical Center, Myrtle Beach, USA

**Keywords:** right atrial thrombus, superior vena cava (svc) obstruction, unexplained syncope, syncope, unusual cause of recurrent syncope, endobronchial ultrasound (ebus), incidental diagnosis, svc syndrome

## Abstract

The superior vena cava (SVC) is mainly responsible for the return of blood flow from the head, upper limbs, and neck into the right atrium. The large vein can be subject to extrinsic tumor compression and invasive intraluminal tumors-metastatic and mediastinal tumors that can lead to complete or partial occlusion. SVC occlusion can also result from chronic inflammation or scarring of the vessel iatrogenically by pacemaker wires or venous access ports used for chemotherapy, long-term antibiotics, or hemodialysis. Patients with SVC occlusion present with a constellation of clinical abnormalities that make up SVC syndrome. SVC syndrome includes varying degrees of facial fullness, neck and upper extremity swelling, dyspnea, and classically dilated collateral veins in the upper chest. Very rarely do patients present with syncope, hoarseness, dysphagia, or acute encephalopathy. The diagnosis of SVC syndrome is best established on imaging such as CT Chest with contrast; however, on rare occasions, it can be discovered by endobronchial ultrasound. We present an unusual presentation of SVC syndrome- primarily presenting as frequent syncopal episodes- diagnosed via endobronchial ultrasound.

## Introduction

Superior vena cava (SVC) syndrome was first defined in 1757 by Scottish surgeon Dr. William Hunter in a case of syphilitic aortitis [1]. With the advent of antibiotics, syphilis no longer accounts for most cases of SVC syndrome. Pathologies that can cause this condition include stenosis of the SVC, external tumors, tumor thrombus, fibrin-derived thrombus, pacemakers, defibrillators, or central venous catheters. There have been increased iatrogenic reasons for SVC syndrome due to central line placements, pacemakers, defibrillators, etc. [1,2]. Malignancies such as small cell lung cancer, non-small cell lung cancer, and non-Hodgkin's lymphoma now account for up to 60 to 85% of SVC syndrome in the United States [1,2]. Most symptoms arise and are due to swelling at the site of a blockage. The degree of symptoms exhibited by the patient is closely correlated with the location of SVC invasion and the degree of occlusion, whether it is a partial versus a complete occlusion. Common symptoms of SVC syndrome include, but are not limited to, face/neck swelling, distended neck veins, cough, dyspnea, orthopnea, upper extremity swelling, distended chest vein collaterals, and conjunctival suffusion [3]. There are rare symptoms, such as syncope and headache, which only occur in approximately 6-13% of cases of SVC syndrome [1].

## Case presentation

This is a case of a 54-year-old male with a medical history significant for chronic obstructive pulmonary disease (COPD) secondary to chronic tobacco abuse who presented with intermittent hemoptysis and cough specifically associated with syncopal episodes. The patient reported that his symptoms began with worsening dyspnea and wheezing over the past five months. About three weeks before the presentation, the patient developed a more productive cough with discolored phlegm, which changed into frank hemoptysis. The patient had been to the emergency department (ED) on two prior occasions for multiple syncopal episodes, worsening dyspnea, chest tightness, and left arm numbness. During those visits, a computed tomography (CT) of the head without contrast was negative for acute intracranial abnormalities. A chest radiograph showed right suprahilar fullness with recommendations for a follow-up chest CT to exclude a mass or adenopathy. The patient was discharged from the ED with antibiotics, nebulized inhalers, and a referral to outpatient pulmonary services. 

The patient returned to the ED a week later as the syncopal episodes were becoming more frequent, occurring sometimes once an hour. An electrocardiogram (ECG) revealed normal sinus with a regular rhythm and a rate of 90 beats per minute. There was no evidence of atrioventricular nodal dissociation in the form of heart blocks, pauses, or skipped beats. The remaining vitals were unremarkable, including orthostatic vitals, metabolic panel, and complete blood count. A repeat chest radiograph was unchanged from the previous radiograph; thus, computed tomography angiography (CTA) of the chest was obtained. The initial impression was without evidence of acute pulmonary embolism but revealed a right hilar mass with conglomerate right paratracheal adenopathy compatible with neoplasm. Scattered nodular and ground-glass airspace diseases in the right upper lobe could be a combination of pneumonitis or hemorrhage. Further recommendations included a bronchoscopy for tissue diagnosis and a positron emission tomography (PET) scan for neoplastic staging. 

The pulmonary service was consulted for bronchoscopy with a tumor biopsy. In the meantime, the patient received treatment for post-obstructive pneumonitis in the inferior right upper lobe. Before performing the bronchoscopy, the pulmonary team reassessed the patient and obtained a detailed history and a thorough review of his chest imaging. His physical exam was remarkable for mild fullness and erythema in the face and neck, but no collateral veins were noted on his chest. This finding, in addition to the recurrent syncope associated with coughing, raised suspicion about SVC syndrome. 

**Figure 1:**
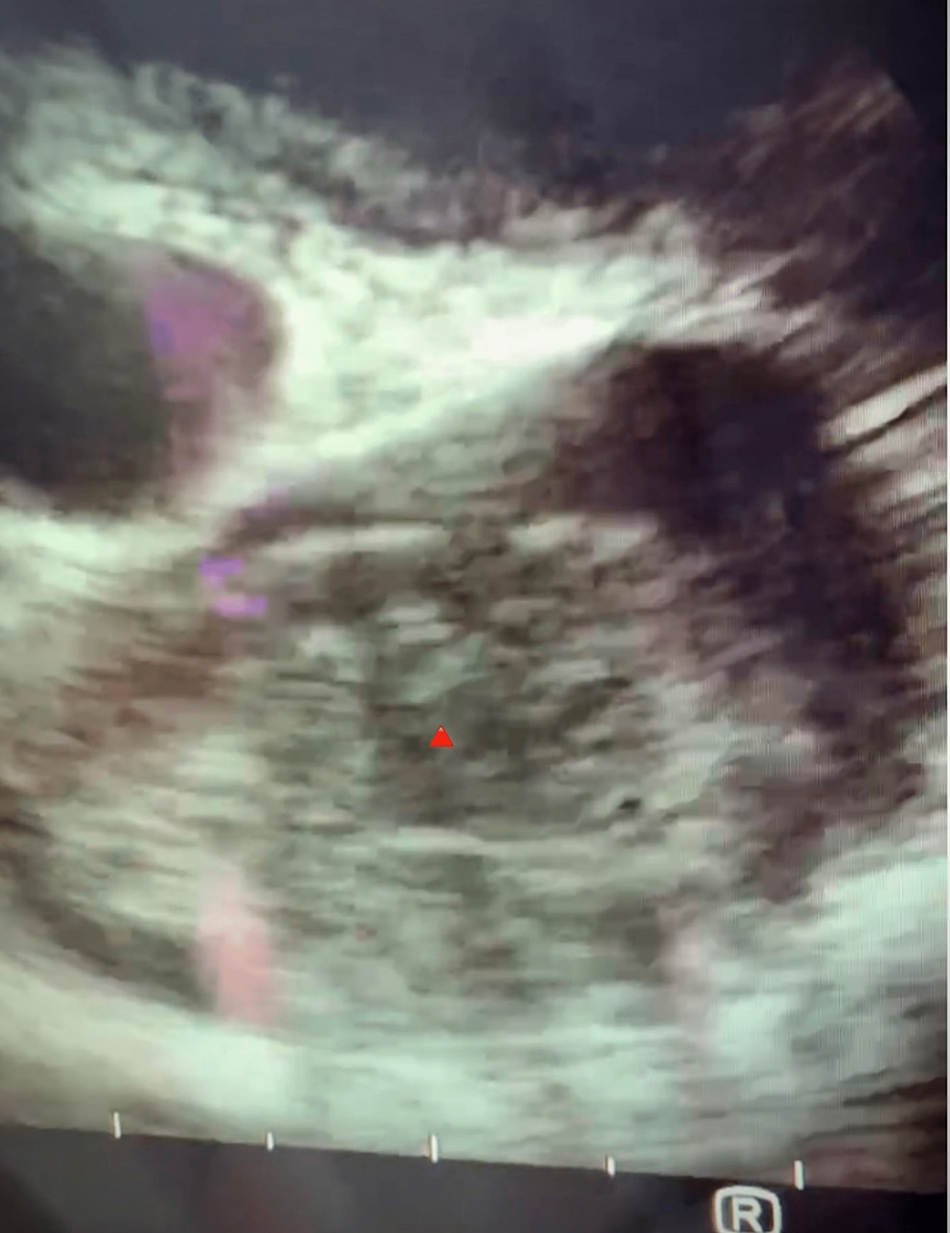
An intraluminal tumor thrombus (red triangle) within the SVC diagnosed by Endobronchial Ultrasound with Trans-bronchial Needle Aspiration (EBUS-TBNA). The patent lumen, posterior to the SVC, is the aorta. SVC- superior vena cava;

An Endobronchial Ultrasound with Trans-bronchial Needle Aspiration (EBUS-TBNA) was performed to aid in further diagnosis. The procedure revealed, in real-time, an intravascular tumor within the lumen of the SVC (Figure 1). It further showed that the trachea appeared to have extrinsic compression distally in the main carina and was splayed in appearance. There were noted to be bloody secretions extending down the right mainstem bronchus. There was a large 4 cm mass at station 4R, also known as the right lower paratracheal lymph nodes. The mass was noted eroding into the SVC and likely azygous vessels. This was biopsied for pathologic analysis and definitive determination of the neoplastic diagnosis.

A post-bronchoscopy CTA Chest with contrast confirmed a definite filling defect within the superior vena cava due to a tumor thrombus extending inferiorly to the level of the right atrial inflow tract.

**Figure 2:**
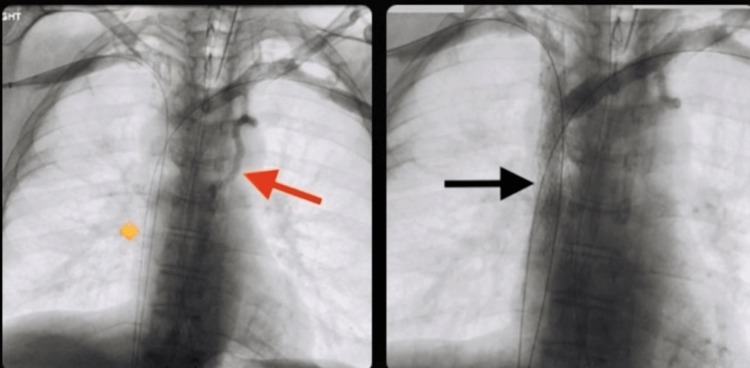
The left image is central venography from bilateral axillary vein injections demonstrating severe SVC stenosis due to a known tumor. The yellow diamond shape is placed where the SVC should be located. However, the Azygous vein (RED arrow) is noted to be more prominent. The right image was taken after percutaneous balloon angioplasty and stent placement which showed significant flow through the SVC without residual stenosis, as seen with the BLACK arrow. Also, note that the Azygous vein is not as prominent with relief of SVC obstruction.

**Figure 3:**
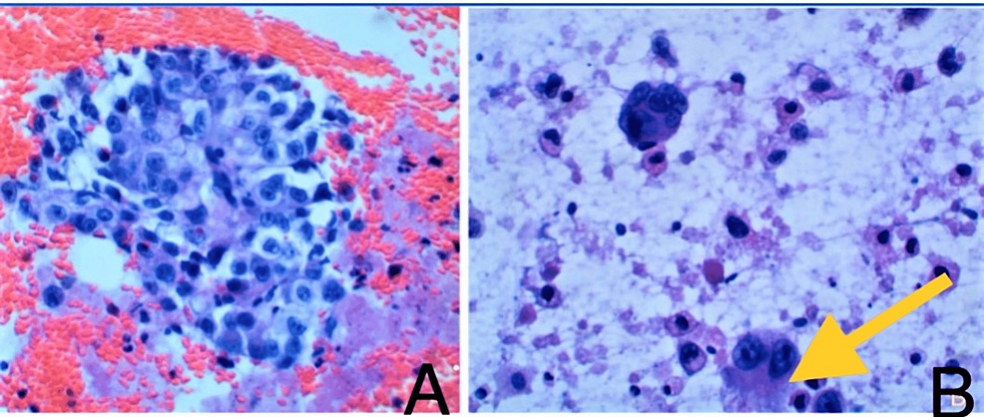
Tumor cells exhibit marked pleomorphism with prominent nucleoli, coarse chromatin, and cellular dyscohesion (Cell block A). The cytoplasm is delicate and foamy as demonstrated by the Yellow arrow), favoring adenocarcinoma over squamous cell carcinoma (Cell block B). Immunohistochemical IHC C analysis revealed a positive nuclear reaction for TTF-1, consistent with pulmonary adenocarcinoma. Thyroid transcription factor 1 (TTF-1) is commonly overexpressed in adenocarcinoma versus non-adenocarcinoma lung tumors.

Management of the SVC syndrome included five fractions of palliative radiation therapy (over two weeks) targeting the mediastinal mass and methylprednisolone 60 milligrams intravenously every 6 hours. After completing radiation therapy, the patient unfortunately left against medical advice but returned a week later with worsening facial swelling and recurrent syncope. At this time, he was no longer a candidate for radiation therapy, and a stent was placed in the SVC (figure 2). This successfully resolved the symptoms of recurrent syncope. Furthermore, the tissue specimens' results for pathology confirmed invasive adenocarcinoma primary to the lung (figure 3).

## Discussion

The superior vena cava comprises the left and right brachiocephalic veins [4]. The SVC is a large valve-less vein that tracks into the superior and middle mediastinum emptying into the right atrium [4,5]. Originally, SVC syndrome was commonly described as secondary to infection [1,2]. However, SVC became associated with malignancy, most commonly primary lung malignancies [6]. Obstruction of the SVC leading to superior vena cava syndrome is an oncological emergency [1]. The period in which obstruction occurs can lead to severe, life-threatening consequences if not recognized immediately.

In some cases, SVC occlusion occurs over a longer time, allowing venous collaterals to aid in drainage back to the heart [5]. The formation of these collaterals is a function of the site of obstruction with the function of the obstruction's position relative to the azygous vein [7]. With the impaired venous return, there is an increase in venous hydrostatic pressure leading to objective abnormalities of facial fullness, upper extremity edema, and engorgement of precordial collateral veins. 

The gold standard for diagnosis of SVC syndrome is a Chest CT Venogram with contrast [1]. Endobronchial ultrasound with trans-bronchial needle aspiration (EBUS-TBNA) is routinely used for sample collection and staging of intrathoracic, especially mediastinal lymph nodes and tumors. There have been case reports of incidental deep vein thrombosis found during EBUS procedures [8]. In managing SVC obstruction, there is limited research to highlight the role of EBUS in prognosis, management, and outcome. One study examining the safety and feasibility of using EBUS-TBNA to aid in the clinical diagnosis of SVC syndrome determined the procedure to be of high diagnostic yield, safe and reliable [9]. Nonetheless, the procedure is invasive and requires anesthesia; it is not routinely used to diagnose SVC obstruction unless the initial diagnosis was not accurately established on a CT Chest with contrast or there is a clear indication to obtain tissue samples for further guidance on medical treatment plans. Both of these were indications for EBUS-TBNA in our patient's presentation. 

Management of SVC syndrome is dependent upon establishing the cause of the obstruction. In general, patients are highly encouraged to elevate the head of the bed in order to reduce the hydrostatic pressure that results from complete obstruction. In malignancy-related SVC obstruction, radiation therapy (RT) used to be the first-line [1]. However, RT can sometimes result in significant edema and can take three days to 3 weeks to provide adequate symptomatic relief [1,2]. In radiation-related edema cases, high-dose corticosteroids should be used to prevent worsening swelling, morbidity, and mortality. Some clinicians will initiate palliative RT alongside targeted chemotherapy or immunotherapy based on pathology results. Recent management of SVC occlusion has shifted from RT as first-line towards Endovascular Therapy (ET) as these interventions provide immediate symptomatic relief with fewer complications [1,2]. Endovascular procedures include catheter-directed thrombolysis, thrombectomy, angioplasty, and stent placement. Antithrombotic therapy, such as aspirin, should be used in cases where stents are used to recanalize the superior vena cava. Anticoagulation, such as apixaban, is recommended in cases where SVC occlusion occurs secondary to fibrinous-rich thrombosis from hypercoagulable states.

## Conclusions

SVC syndrome is a constellation of symptoms resulting from intrinsic or extrinsic flow obstruction through the superior vena cava. It has been established as an oncologic emergency that can result in significant facial swelling, airway obstruction, coma, and death. Diagnosis is achieved with a high degree of clinical suspicion and advanced imaging such as a CT Chest with contrast. Endobronchial Ultrasound with Trans-bronchial Needle Aspiration (EBUS-TBNA) provides high diagnostic yield; however, it is an invasive procedure requiring anesthesia and thus must be discussed with the patient as part of a shared decision-making process, especially if there is a higher clinical benefit in proceeding with an EBUS. Direct pathologic samples obtained by TBNA can be used to identify the cause of the occlusion and simultaneously stage mediastinal tumors. Furthermore, although recurrent syncope is not a common symptom of this syndrome, recurrent syncopal episodes with coughing should trigger an investigation for SVC occlusion in the differential diagnosis.
